# Supplementary material for: FastqPuri: high-performance preprocessing of RNA-seq data
Source: BMC Bioinformatics. 2019 May 3;20:226. doi: 10.1186/s12859-019-2799-0 (PMC6500068; doi:10.1186/s12859-019-2799-0)
Supplement: Supplementary file 2 — Archive of FastqPuri. Archive containing all files needed to install and run FastqPuri v1.0.6. Date stamp March 22, 2019. (GZ 47,819 kb) [file 12859_2019_2799_MOESM2_ESM.gz › FastqPuri-1.0.6/html/io__trimFilter_8h_source.html]

FastqPuri: include/io\_trimFilter.h Source File


FastqPuri


- include

io\_trimFilter.h

Go to the documentation of this file.

1 /\*\*\*\*\*\*\*\*\*\*\*\*\*\*\*\*\*\*\*\*\*\*\*\*\*\*\*\*\*\*\*\*\*\*\*\*\*\*\*\*\*\*\*\*\*\*\*\*\*\*\*\*\*\*\*\*\*\*\*\*\*\*\*\*\*\*\*\*\*\*\*\*\*\*\*\*

2  \* Copyright (C) 2017 by Paula Perez Rubio \*

3  \* \*

4  \* This file is part of FastqPuri. \*

5  \* \*

6  \* FastqPuri is free software: you can redistribute it and/or modify \*

7  \* it under the terms of the GNU General Public License as \*

8  \* published by the Free Software Foundation, either version 3 of the \*

9  \* License, or (at your option) any later version. \*

10  \* \*

11  \* FastqPuri is distributed in the hope that it will be useful, \*

12  \* but WITHOUT ANY WARRANTY; without even the implied warranty of \*

13  \* MERCHANTABILITY or FITNESS FOR A PARTICULAR PURPOSE. See the \*

14  \* GNU General Public License for more details. \*

15  \* \*

16  \* You should have received a copy of the GNU General Public License \*

17  \* along with FastqPuri. \*

18  \* If not, see <http://www.gnu.org/licenses/>. \*

19  \*\*\*\*\*\*\*\*\*\*\*\*\*\*\*\*\*\*\*\*\*\*\*\*\*\*\*\*\*\*\*\*\*\*\*\*\*\*\*\*\*\*\*\*\*\*\*\*\*\*\*\*\*\*\*\*\*\*\*\*\*\*\*\*\*\*\*\*\*\*\*\*\*\*\*\*/

20

29 #ifndef IO\_TRIMFILTER\_H\_

30 #define IO\_TRIMFILTER\_H\_

31

32 #include <stdio.h>

33 #include "defines.h"

34

38 typedef struct \_stats\_TF {

39  int filters[NFILTERS];

40  int trimmed[NFILTERS];

41  int discarded[NFILTERS];

42  int good;

43  int nreads;

44 } Stats\_TF;

45

46 void buffer\_output(FILE \*fout, const char \*a, const int len, const int fd\_i);

47

48 void write\_summary\_TF(Stats\_TF tf\_stats, char \*filename);

49

50 #endif // IO\_TRIMFILTER\_H\_

\_stats\_TF::nreads

int nreads

**Definition:** io\_trimFilter.h:43

\_stats\_TF::filters

int filters[NFILTERS]

**Definition:** io\_trimFilter.h:39

\_stats\_TF

collects stats info from the filtering procedure

**Definition:** io\_trimFilter.h:38

\_stats\_TF::trimmed

int trimmed[NFILTERS]

**Definition:** io\_trimFilter.h:40

defines.h

Macro definitions.

NFILTERS

#define NFILTERS

**Definition:** defines.h:128

write\_summary\_TF

void write\_summary\_TF(Stats\_TF tf\_stats, char \*filename)

writes stats of filtering to summary file (binary)

**Definition:** io\_trimFilter.c:62

\_stats\_TF::discarded

int discarded[NFILTERS]

**Definition:** io\_trimFilter.h:41

buffer\_output

void buffer\_output(FILE \*fout, const char \*a, const int len, const int fd\_i)

buffers the output before writing to disk, writes out summary

**Definition:** io\_trimFilter.c:44

\_stats\_TF::good

int good

**Definition:** io\_trimFilter.h:42

Stats\_TF

struct \_stats\_TF Stats\_TF

collects stats info from the filtering procedure


---

Generated on Mon Mar 19 2018 23:42:01 for FastqPuri by  

 1.8.14
